# Malaria treatment in the retail sector: Knowledge and practices of drug sellers in rural Tanzania

**DOI:** 10.1186/1471-2458-8-157

**Published:** 2008-05-09

**Authors:** Manuel W Hetzel, Angel Dillip, Christian Lengeler, Brigit Obrist, June J Msechu, Ahmed M Makemba, Christopher Mshana, Alexander Schulze, Hassan Mshinda

**Affiliations:** 1 Dept. of Public Health and Epidemiology, Swiss Tropical Institute, P.O. Box, CH-4002 Basel, Switzerland; 2 Ifakara Health Research and Development Centre, P.O. Box 53, Ifakara, Tanzania; 3 Novartis Foundation for Sustainable Development, WRO-1002.11.56, CH-4002 Basel, Switzerland

## Abstract

**Background:**

Throughout Africa, the private retail sector has been recognised as an important source of antimalarial treatment, complementing formal health services. However, the quality of advice and treatment at private outlets is a widespread concern, especially with the introduction of artemisinin-based combination therapies (ACTs). As a result, ACTs are often deployed exclusively through public health facilities, potentially leading to poorer access among parts of the population. This research aimed at assessing the performance of the retail sector in rural Tanzania. Such information is urgently required to improve and broaden delivery channels for life-saving drugs.

**Methods:**

During a comprehensive shop census in the districts of Kilombero and Ulanga, Tanzania, we interviewed 489 shopkeepers about their knowledge of malaria and malaria treatment. A complementary mystery shoppers study was conducted in 118 retail outlets in order to assess the vendors' drug selling practices. Both studies included drug stores as well as general shops.

**Results:**

Shopkeepers in drug stores were able to name more malaria symptoms and were more knowledgeable about malaria treatment than their peers in general shops. In drug stores, 52% mentioned the correct child-dosage of sulphadoxine-pyrimethamine (SP) compared to only 3% in general shops. In drug stores, mystery shoppers were more likely to receive an appropriate treatment (OR = 9.6), but at an approximately seven times higher price. Overall, adults were more often sold an antimalarial than children (OR = 11.3). On the other hand, general shopkeepers were often ready to refer especially children to a higher level if they felt unable to manage the case.

**Conclusion:**

The quality of malaria case-management in the retail sector is not satisfactory. Drug stores should be supported and empowered to provide correct malaria-treatment with drugs they are allowed to dispense. At the same time, the role of general shops as first contact points for malaria patients needs to be re-considered. Interventions to improve availability of ACTs in the retail sector are urgently required within the given legal framework.

## Background

Treatment-seeking behaviour for malaria in sub-Saharan Africa is complex, often involving several steps and actors, depending on the local health system, society and culture [1,2]. As a result of poor access to and often poor performance of formal health services, presumptive treatment of malaria episodes at home has become a widespread option [3,4]. The home-management of malaria (HMM) strategy of the WHO is promoting interventions to improve antimalarial drug use outside the formal health services as a complementary option to improve access to prompt and effective treatment at community level [5].

In most places, the private retail sector has been identified as an important source of drugs close to people's homes [6-8]. However, the regimens dispensed by private retailers are often inadequate with regard to the type of drug and their dosage [9-11]. In order to increase community-wide effectiveness of antimalarial treatment, the popularity of home-management and the quality of treatment obtained from commercial shops need to be better addressed. Considerable improvement in case-management has been shown to be possible as a result of training private retailers in general shops [12] and in drug stores [13].

**Figure 1:**
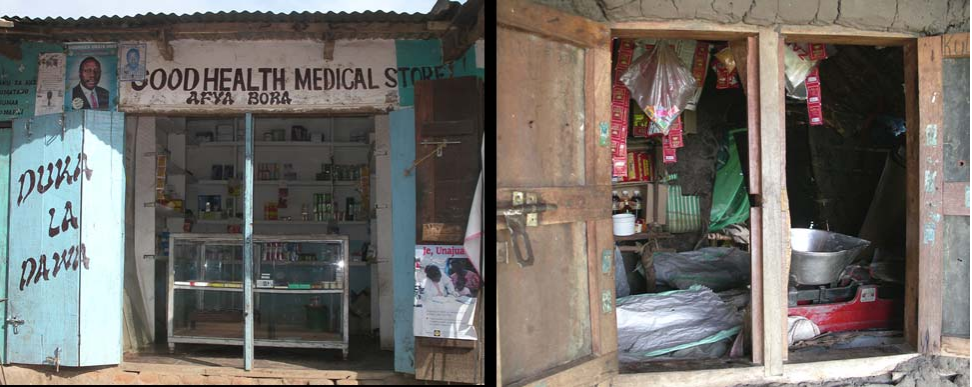
**Examples of a part II drug store (left) and a general shop (right)**. Both shop types are providers of malaria treatment in rural Tanzania.

In Tanzania, the private retail sector plays a central role in the provision of malaria treatment, partly complementing health facility services where these are unable to deliver [14]. In rural areas, 68% of the population live within 5 km of a health centre or a dispensary (98% in urban areas) [15]. Yet, poor quality of care, shortage of skilled providers, stock-outs of essential drugs, and long waiting times [16,17] may drive patients to seek care (or at least buy drugs) from more expensive non-governmental facilities, or from shops. The Tanzanian retail sector for drugs includes two types of licensed drug stores as well as general shops. Fully-fledged pharmacies are allowed to sell all prescription medicines and need to be headed by a pharmacist. In 2003, 60% of the 344 existing pharmacies were located in Dar es Salaam and the rest in other larger towns [18]. Part II drug stores (in Swahili: *Duka la Dawa Baridi) *need to be headed by a person with basic medical or health-related training and can be found in towns and larger villages. Part II shops are allowed to sell over-the-counter (OTC) drugs only (e.g. analgesics/antipyretics). In practice however, they dispense a much wider variety of medicines. This usually includes certain antimalarials, all of which are prescription-only medicines (except for oral amodiaquine) [19]. In 2003, 5666 registered part II drug stores were operating in Tanzania [18]. The legal situation regarding drug sales in general shops was unclear [20]. It appeared that, while they were not allowed to stock any drugs, they were often selling common OTC medicines, such as painkillers (Figure 1).

The studies presented here made use of selected key indicators in a mixed methods approach to compare factual knowledge with every-day practices of private drug retailers in treating cases of malaria in two Tanzanian districts. The research aimed to provide an assessment of the quality of malaria case-management in shops in order to inform interventions targeted at the retail sector. We included retailers in drug stores as well as general shops in order to get a comprehensive picture of the quality of treatment and advice that can be obtained from shops. This information is particularly important in the light of ongoing discussions on suitable distribution channels for artemisinin-based combination therapies (ACT). The studies were carried out within the frame of a project to improve access to prompt and effective malaria treatment in rural Tanzania (ACCESS Programme) [21].

## Methods

### Study setting

A systematic shop census and a complementary study using mystery shoppers were conducted in the districts of Kilombero and Ulanga, Morogoro Region, south-eastern Tanzania. The study area comprised the 25 villages of the local Demographic Surveillance System (DSS) [22] and the town of Ifakara, 20 km to the east of the DSS. The mid-2004 population of the DSS was 74,200 and Ifakara had a population of 45,726 in the 2001 population census [23].

Malaria is highly endemic in the area, accounting for roughly half of all outpatient visits in rural health facilities. The study area is described in more detail elsewhere [21]. Previous studies in the same setting found a range of easily accessible commercial outlets frequently selling drugs for fever episodes [8]. In 2004, 29 part II drug stores and 460 general shops stocking drugs were counted and chloroquine was found to be completely replaced on the market by sulphadoxine-pyrimethamine (SP) and amodiaquine [24].

At the time of the surveys, SP was the recommended first-line treatment for uncomplicated malaria; amodiaquine and quinine were second-line and third-line treatment, respectively. Quinine was the drug of choice for severe malaria [25]. All antimalarials were prescription-only medicines and could therefore legally be sold only in the one registered pharmacy located in Ifakara town. However, part II drug stores which were found in some villages were generally tolerated to stock and sell antimalarials. General shops were not allowed to stock any prescription drugs, which was reflected in the low availability of antimalarials reported elsewhere [24].

### Shop census

Between May and June 2004, all commercial outlets in the DSS area and Ifakara town were visited in order to investigate the availability of antimalarial drugs in the retail sector. The detailed methodology of this census, as well as the results on drug availability have been published elsewhere [24]. This paper makes use of additional information on shopkeepers' knowledge of malaria and its treatment, collected during the same survey. Interviews were carried out with shopkeepers or acting drug vendors if the shopkeepers were not present. They were asked to name signs and symptoms of "malaria" and to explain the recommended treatment of "uncomplicated malaria" in children of two years of age and adults. We used the terms "malaria" and "uncomplicated malaria" (in Swahili: *malaria isiyo kali*) in the same was as they were used in information materials produced by the National Malaria Control Programme. In addition, the interviewers recorded information on the estimated number of customers per day.

### Mystery shoppers

The results of the census were complemented in September and October 2004 by "mystery shoppers", simulated clients who purchased drugs for predefined malaria case-scenarios.

From a preliminary list of outlets stocking drugs in 2004 (n = 510), a sample of approx. 20% (111) of all general shops was chosen at random. The sample size was defined mainly on the basis of operational considerations. The sample was drawn per village and weighed by village size. A back-up sample was drawn to compensate for shops that would be closed or could not be visited for other reasons. In addition, all 19 drug stores from the DSS area and 10 from Ifakara town were added to the sample.

One of the following three case scenarios was randomly assigned to each of the sampled shops.

(A) child aged 2–4 months, with fever/hot body for one day and problems with drinking/breastfeeding

(B) child aged 2–4 years, with recurring fever/hot body for 3 days (especially at night), problems with drinking and eating, diarrhoea and tiredness/not playing as usual

(C) adult, with recurring fever/hot body for 2 days, headache, dizziness and loss of appetite.

The scenarios were developed based on the list of common signs and symptoms of mild malaria in the guidelines of the National Malaria Control Programme [25]. All scenarios did explicitly exclude signs of convulsions or unconsciousness, which would be an indication for severe disease. Mystery shoppers were trained to report only the above listed symptoms to the vendors in the shops. For the child-scenarios, the mystery shoppers would carry their children when visiting the shops, if at all possible.

Local DSS field staff recruited mystery shoppers from the villages in which the respective shops were located. On the day of the study, the mystery shoppers were trained by project staff on how to approach a shop, and which symptoms to report or not report. Mystery shoppers were asked to visit one selected shop and ask for treatment based on the aforementioned case-scenario. Each mystery shopper received 2,000 Tanzanian shilling (TSh) (US $1.80) to buy drugs. After completing their assignment, they were interviewed by project staff about what exactly happened when they visited the shops, what they had told the shopkeeper, and what advice and drugs they were given. Interviews were tape-recorded and later transcribed. Drugs and remaining money were collected, types and amount of drugs recorded, and the mystery shoppers were paid a small allowance for their collaboration.

### Data entry and analysis

Generic and brand names (if possible), as well as amount and price of the drugs obtained by the mystery shoppers were entered in a Microsoft Access database (Microsoft Corp., Seattle, USA). Interviews with the mystery shoppers were entered with word processing software in an RTF file and imported into MAXqda software (VERBI GmbH, Marburg, Germany) for coding of the answers. Statistical analysis was done with Intercooled Stata 9 (StataCorp, College Station, Texas, USA).

### Ethics

While mystery shoppers were fully informed and asked for informed consent, the nature of this study did not allow informing the shopkeepers in advance and asking them for consent to participate. To protect shopkeepers' privacy, no names of staff were recorded and names of shops were never mentioned in connection with the study's results. For the shop census, informed consent was obtained from shopkeepers as described in detail in the aforementioned publication.

The shop survey and mystery shopper study were granted ethical clearance as part of the ACCESS Programme proposal by the institutional review board of the Ifakara Health Research and Development Centre and the Tanzanian National Medical Research Coordinating Committee (NIMR/HQ/R.8a/Vol.IX/236).

## Results

### Shop census

The sample for this analysis included interviews with shopkeepers of 29 part II drug stores and 460 general shops, all of which stocked drugs the day of the interview. General shopkeepers had on average a lower education than their peers in drug stores (7 vs.10 years, P < 0.001). A shopkeeper with medical or health-related qualifications was found in 93% of the drug stores and 2% of the general shops (P < 0.001). Shopkeepers reported the number of customers buying drugs per day to be on average 19 (95% CI 14–24) in drug stores and 10 (9–11) in general shops (P < 0.001).

### Knowledge of malaria symptoms and treatment

Shopkeepers of drug stores most frequently mentioned fever, headache and vomiting (86% each) as symptoms of malaria (not specified whether in children or adults). In general shops, fever (60%), headache (40%) and joint pains (39%) were most frequently mentioned (Table 1). Generally, shopkeepers of general shops seemed to be significantly less aware of malaria symptoms. They mentioned all of the recorded symptoms less frequently than shopkeepers of drug stores. Out of 15 symptoms associated with malaria, shopkeepers in drug stores mentioned on average 4.8 (95% CI 4.1 to 5.5), while in general shops they mentioned only 2.4 (2.3–2.5) (P = 0.005). If asked for "severe malaria" (in Swahili: *malaria kali*), a similar picture arose. The symptoms most often mentioned by general shopkeepers were high fever (44%) and weakness (18%), while in drug stores, shopkeepers most often mentioned high fever (79%) and convulsions (*degedege*) (52%) (Table 1).

**Table 1:** Malaria symptoms mentioned most frequently by shopkeepers (N = 489)

	**Drug store**	**General shop**	
		
**Symptom**	**% (95% CI)**	**% (95% CI)**	P*
	
N	29	460	
**What are symptoms of malaria?**			

Fever	86 (68–96)	60 (56–65)	**0.006**
Headache	86 (68–96)	40 (36–45)	**0.000**
Joint pains	62 (42–79)	39 (34–44)	**0.014**
Vomiting	86 (68–96)	33 (28–37)	**<0.001**
Malaise	31 (15–51)	20 (16–24)	0.138
Feeling cold	17 (6–36)	16 13–20)	0.895
Poor appetite	21 (8–40)	10 (7–13)	0.063
Weakness	28 (13–47)	7 (5–10)	**<0.001**
Diarrhoea	28 (13–47)	6 (4–8)	**<0.001**
Dizziness	14 (4–32)	4 (2–6)	**0.013**
Don't know	0 (0–12)	12 (9–15)	0.053

**What are symptoms of severe malaria?**			

Changed behaviour	24 (10–44)	17 (14–21)	0.326
Unconsciousness/coma	17 (6–36)	7 (5–10)	0.059
Weakness	35 (18–54)	18 (15–22)	**0.027**
Anaemia	10 (2–27)	1 (0–2)	**<0.001**
Convulsions (*degedege*)	52 (33–71)	10 (7–13)	**<0.001**
Splenomegaly (*bandama*)	3 (0–18)	0 (0–1)	**0.008**
High fever	79 (60–92)	44 (39–38)	**<0.001**
Don't know	3 (0–18)	27 (23–32)	**0.005**

* Wilcoxon rank sign test

In drug stores, most shopkeepers knew that an antimalarial drug was the recommended treatment for malaria in a two year-old child (90%) and in an adult (93%). In general shops, shopkeepers most frequently said that the child should be referred to a health facility (34%) while adults should take an antimalarial drug (54%). Shopkeepers of drugs stores had significantly better knowledge of malaria treatment, as shown in Table 2. In drug stores, 66% mentioned SP as the recommended treatment for a child aged two years and 79% for an adult. In general shops this percentage was significantly lower. Of those who mentioned SP, 79% (54–94) knew the correct child dose in drug stores and 27% (16–40) in general shops (P < 0.001). No shopkeeper mentioned traditional treatment, or that the episode should not be treated at all.

**Table 2:** Shopkeepers' understanding of the recommended treatment of uncomplicated malaria (N = 489)

	**Drug store**	**General shop**	
		
**Treatment^‡^**	**% (95% CI)**	**% (95% CI)**	P*
	
N	29	460	
**Child aged two years with uncomplicated malaria**			

Referral to health facility	3 (0–18)	34 (30–39)	**0.001**
Antipyretic	55 (36–74)	31 (27–35)	**0.007**
Antimalarial	90 (73–98)	32 (28–36)	**<0.001**
- SP	66 (46–82)	12 (9–16)	**<0.001**
- SP + PCM	35 (18–54)	5 (3–7)	**<0.001**
- SP correct dose	52 (33–71)	3 (2–5)	**<0.001**
- SP correct dose + PCM	31 (15–51)	1 (0–2)	**<0.001**

**Adult with uncomplicated malaria**			

Referral to HF^†^	0 (0–12)	24 (20–28)	**0.003**
Antipyretic	55 (36–74)	44 (39–49)	0.237
Antimalarial	93 (77–99)	54 (49–58)	**<0.001**
- SP	79 (60–92)	35 (31–40)	**<0.001**
- SP + PCM	48 (29–68)	16 (13–19)	**<0.001**
- SP correct dose	76 (57–90)	29 (25–33)	**<0.001**
- SP correct dose + PCM	45 (26–64)	14 (11–18)	**<0.001**

SP = Sulphadoxine-pyrimethamine; PCM = Paracetamol.

* Wilcoxon rank sign test.

^‡ ^Double-mentioning possible.

^† ^one-sided, 97.5% confidence interval.

In a multivariate logistic regression analysis adjusted for shop type, number of customers and shop location, higher general education was a significant predictor of knowing SP as recommended treatment for adults (OR = 1.15, 95% CI 1.02–1.30; P = 0.020). A health-related qualification was a strong predictor of knowing SP as a child treatment (OR = 12.36, 2.45–62.20; P = 0.002). Correctly dosed SP for adults – but not for children – was correlated with higher education (OR = 1.15, 1.01–1.30; P = 0.032) and a health-related qualification (OR = 4.80, 1.08–21.34; P = 0.039). Generally, there seemed to be better knowledge of the appropriate treatment among shopkeepers in Ulanga DSS villages, compared to Kilombero DSS and Ifakara town.

### Referral

Shopkeepers were asked for situations in which they would refer a customer to another outlet or a health facility. In drug stores, 19 or 66% (46–82) of the shopkeepers said they would refer customers if they showed signs of severe malaria, in general shops this was indicated by 259 or 56% (52–61). Of the general shopkeepers, 58 or 13% (10–16) said they would never refer somebody to another outlet or a health facility, while this was never mentioned by shopkeepers of drug stores.

### Mystery shoppers

**Figure 2:**
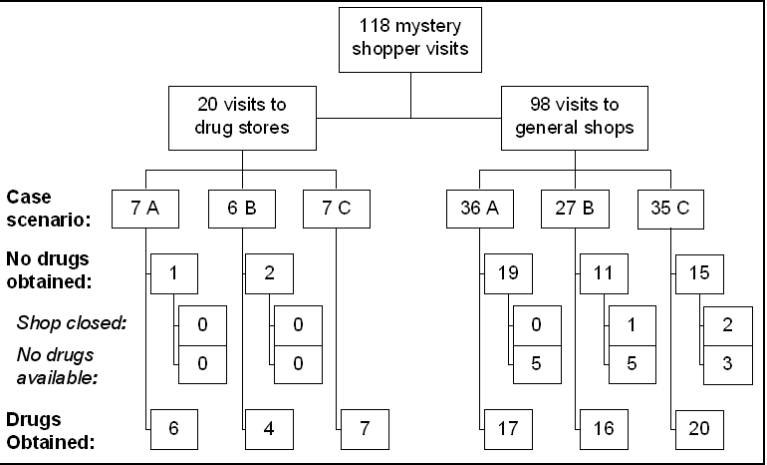
**Flow-chart of mystery shoppers study**. Case scenarios: A = child, aged 2–4 months; B = child, aged 2–4 years; C = adult. Refer to main text for details.

A total of 20 part II drug stores and 98 general shops were visited by mystery shoppers. General shops comprised all sorts of outlets, from permanent modern shops to temporary stalls. Case-scenarios were distributed as shown in Figure 2.

### Drug sale

Mystery shoppers obtained drugs in 53 (54%, 95% CI 62–97) general shops and 17 (85%, 44–64) drug stores (P = 0.010) (Table 3).

**Table 3:** Number of shops that dispensed drugs to mystery shoppers

	**Drug store**	**General shop**	**Total**
	
**Area**	**n/N (%)**	**n/N (%)**	**n/N (%)**
Ulanga DSS	2/2 (100)	23/30 (77)	25/32 (78)
Kilombero DSS	10/10 (100)	20/44 (46)	30/54 (56)
Ifakara	5/8 (63)	10/24 (42)	15/32 (47)

**Total**	**17/20 (85)**	**53/98 (54)**	**70/118 (59)**

Out of the 17 **drug stores **that sold drugs, 88% (64–99) dispensed an antipyretic and the same percentage an antimalarial (Table 4). SP and amodiaquine were sold most often. Antimalarials were usually sold together with paracetamol, a practice which is recommended for SP in the national guidelines [25].

**Table 4:** Types of medicines sold to mystery shoppers

	**Drug stores**		**General shops**	
N	17		53	

**Type of drugs**	**n**	**%**	**n**	**%**

**Antimalarials**				
SP	6	35	5	9
Amodiaquine	6	35	4	8
Quinine	4	24	0	
Any antimalarial	15	88	10	19

**Antipyretics**				
Paracetamol	14	82	39	74
Any antipyretic	15	88	45	85

**Other drugs**				
Antibiotic	2	12	2	4
Vitamin B complex	5	29	0	

**Combinations**				
SP & paracetamol	6	35	3	6
Amodiaquine & paracetamol	4	24	2	4
Quinine & paracetamol	4	24	0	
SP & quinine	1	6	0	
Antimalarial & antibiotic	2	12	0	

No antimalarials other than SP, amodiaquine or quinine were dispensed and only two drug stores sold an antibiotic. In the Kilombero DSS villages, Vitamin B was sometimes dispensed together with antimalarials.

Of the 53 **general shops**, 85% (72–93) sold an antipyretic, usually paracetamol and 19% (9–32) sold an antimalarial, either SP or amodiaquine. About half of the antimalarial dosages were sold together with paracetamol. Two general shop sold an antibiotic (Table 4).

On average, drug stores sold more products per client than general shops, which often had only paracetamol on offer. Drug stores most frequently sold two products (to 44% of the mystery shoppers) (mean 2.9 products, 95% CI 2.0 to 3.8). General shops most often sold only one product (66% of the mystery shoppers) (mean 1.5 products, 1.2 to 1.8).

### Predictors of drug sale

Univariate and multivariate models were fitted to assess factors related to obtaining an antimalarial and obtaining an antimalarial treatment according to Tanzanian guidelines.

Adjusted for the confounding effect of age group (i.e. case scenarios A, B, C, as described above), visits to a drug store resulted significantly more often in obtaining a drug than visits to a general shop (OR = 6.02, 95% CI 1.57–23.10) and shopkeepers in the DSS were more likely to sell a drug than their counterparts in Ifakara (OR = 2.53, 1.04–6.18).

In drug stores, mystery shoppers were significantly more likely to receive an antimalarial (OR = 76.47, 13.07–447.50) (Table 5). Adults were more likely to be sold an antimalarial compared with infants (OR = 9.30, 1.70–50.92) and compared with the two child scenarios (OR = 11.27, 2.36–53.81) (not in table). There was no significant difference in this outcome between shops located in the villages or in Ifakara town.

**Table 5:** Univariate and multivariate logistic regression analysis of the relationship between (any) antimalarial drug obtained and selected predictors (all visited shops)

		**Univariate model**		**Multivariate model**	
		
**Predictor**	**n**	**Odds Ratio (95% CI)**	P*	**Odds Ratio (95% CI)**	P*
**Case scenario**					
- Child 2–4 months	43	1		1	
- Child 1–4 years	33	0.85 (0.22–3.30)	0.815	0.62 (0.09–4.00)	0.612
- Adult	42	3.43 (1.18–9.98)	0.024	9.30 (1.70–50.92)	**0.010**

**Shop type**					
- General shop	98	1		1	
- Drug store	20	26.40 (7.91–88.10)	<0.001	76.47 (13.07–447.50)	**<0.001**

**Location**					
- Ifakara	32	1		1	
- DSS	86	0.95 (0.35–2.53)	0.911	1.65 (0.41–6.66)	0.480

* Wald test of significance of effect

In order to assess whether the observed difference in antimalarial dispensing was due to a lower availability of drugs in general shops, the same analysis was carried out only with shops that had dispensed any drugs at all. It resulted that drug stores were again more likely to dispense an antimalarial than general shops (OR = 70.71, 9.38–533.10). If a drug was sold, mystery shoppers were in both types of shops equally likely to receive an antipyretic drug.

Adjusted for the same confounders as listed in Table 5, mystery shoppers visiting a drug store were more likely to receive the recommended first-line antimalarial SP (OR = 9.62; 1.53–60.53) or even SP together with paracetamol (OR = 16.40; 2.28–117.99) than those who went to a general shop.

Again, the same analysis was carried out only for those shops that had dispensed an antimalarial. In this case, drug stores did not dispense SP (or SP with paracetamol) more often than general shops.

### Price

In drug stores, mystery shoppers paid a median price of TSh 1000 or US $0.90 (interquartile range [IQR] 0.50–1.53) for drugs, while in general shops they spent only TSh 140 or US $0.13 (IQR 0.09–0.29, equality-of-medians test P < 0.001).

In a multivariate linear regression model we assessed the effect of the age group (case scenario A, B, C), the number of products sold, the shop type and the location (Ifakara vs. DSS) on the price charged to the mystery shoppers. Significantly less (-25%) money was spent for adult cases (case scenario C) compared to children aged 2–4 months (case scenario A) (P = 0.012) and 59% more in drug stores than in general shops (P < 0.001). Obviously, more money was spent if more drugs were sold (12% more per additional product; P = 0.001) (Table 6).

**Table 6:** Linear regression model of predictors of higher expenditures for antimalarial drugs

		**Univariate model**		**Multivariate model**	
			
**Risk factors**	**n**	**Estimated effect (95% CI)**	P*	**Estimated effect (95% CI)**	P*
**Case scenario**					
- Child 2–4 months	20	1		1	
- Child 1–4 years	22	-0.12 (-0.42 to 0.18)	0.423	-0.16 (-0.37 to 0.05)	0.124
- Adult	26	-0.15 (-0.44 to 0.13)	0.285	-0.25 (-0.45 to -0.06)	**0.012**

**Number of products**	68	0.19 (0.11 to 0.27)	<0.001	0.12 (0.05 to 0.19)	**0.001**
**Shop type**					
- General shop	50	1		1	
- Drug store	18	0.75 (0.56 to 0.95)	<0.001	0.59 (0.38 to 0.80)	**<0.001**

**Location**					
- Ifakara	15	1		1	
- DSS	53	-0.11 (-0.39 to 0.17)	0.434	-0.06 (-0.24 to 0.13)	0.548

*Wald test of significance of effect

### Dosage and advice

The accuracy of the dosages was judged from the amount of drugs the mystery shoppers obtained and from their accounts of the advice they were given by the shopkeepers.

10/11 (91%) SP doses were tablets, one was a suspension. 4/10 (40%) amodiaquine doses were tablets and 6 were suspensions. Quinine was sold 2/4 (50%) times as tablets, and twice as syrup.

10/11 (91%) SP dosages (incl. the suspension) and 4/10 (40%) amodiaquine dosages (2 tablets, 2 suspensions) were correct, considering the amount sold and the advice given. For two amodiaquine doses, no dosage information was available. Quinine tablets and syrup doses were all wrongly dosed. With the low number of samples no relevant comparison could be made between the appropriateness of the dosages and the shop types. Yet it should be noted that in general stores, all SP dosages tablet were correct, while in the drug stores, 1/6 was under-dosed (adult case). On the other hand, all amodiaquine dosages which were sold in drug stores (and for which the dosage information was available) were correct while those sold in general shops were under-dosed.

3 (15%) drug stores and 29 (30%) general shops did not sell any drugs to the mystery shoppers although they would have had drugs in stock (Figure 1). In all of these drug stores the mystery shoppers were advised to seek treatment or advice from a health facility. In the general shops, 86% (25/29) of the shopkeepers referred the mystery shoppers to a higher level: 62% (18/29) to a health facility and 31% (9/29) to a drug store (some of them to both).

## Discussion

The private retail sector plays a central role in the provision of malaria treatment in Tanzania. In rural areas, 68% of the population live within 5 km of a health centre or a dispensary (98% in urban areas) [15]. Yet, poor quality of care, shortage of skilled providers, stock-outs of essential drugs, and long waiting times [16,17] are challenges which may drive patients to seek care (or at least buy drugs) from more expensive non-governmental facilities, or from drug stores. The private retail sector may complement health facility services where the facilities are unable to deliver [14].

In the studies presented here, drug stores were more frequently visited for drugs than general shops. In an earlier study, general shops have been described as being important treatment sources for fever/malaria, with 29% of fever cases using this source of treatment. Yet, in terms of drug volumes, general shops accounted for only 6–7% of all antimalarial doses dispensed in the two study districts [20]. However, general shops are important first contact points of patients with a network of treatment providers. They are numerous even in small villages and often more easily accessible than drug stores or health facilities [24]. While not being legally allowed to dispense antimalarial drugs, they are recognised in the national policy as one component of the health care delivery structure [25,26]. Yet, their relatively poor knowledge of malaria and its appropriate treatment supports the ban of antimalarial drugs from these outlets. Surprisingly, only 60% of general shopkeepers mentioned *homa *(fever) as a symptom of malaria. In part, this may be explained by the parallel use of *homa *as a term to describe a less severe febrile illness or general malaise [27,28]. Knowing the correct treatment was clearly a function of the shopkeeper's education, which in general shops was lower than in drug stores. However, general shopkeepers did not seem to be completely unaware of their limitations, as 34% of them mentioned referral to a health facility as the correct action for a child with malaria.

Drug stores on the other hand are the lowest level of providers which is generally tolerated to dispense prescription-only antimalarial drugs. Unfortunately, they often do not reach out into small villages or remote areas [24]. Shopkeepers in drug stores were more knowledgeable about malaria-related symptoms and malaria treatment than their counterparts in general shops. This was correlated with basic medical or health-related training, a prerequisite for shopkeepers of licensed part II drug stores [29]. Nevertheless, their performance was not satisfactory, with only 52% mentioning SP in the correct dosage as recommended treatment for children.

### Knowledge vs. practice

In order to get a realistic picture of drug-sellers' performance, we used mystery shoppers; an approach which has been applied frequently in market research, but rarely in a public health context [30,31]. The main challenge of applying this methodology in a rural setting, which is to find capable mystery shoppers within a certain village, was tackled with the help of knowledgeable village-based DSS field staff.

Daily shopkeepers' practices clearly reflected their level of understanding of appropriate treatment, the current drug regulations, as well as the low antimalarial availability in general shops [24]. Antipyretics were frequently sold in both, drug stores and general shops. Most drug stores (88%) also sold antimalarials to the mystery shoppers. In contrast, during a study conducted elsewhere in Tanzania in which shopkeepers were under observation, only 17% of febrile patients had received an antimalarial [9]. In general shops, 19% of the mystery shoppers were sold an antimalarial, which was more than expected based on the shop census in which 8% of all general shops that had drugs in stock also stocked an antimalarial [24].

While many shopkeepers in drug stores knew that SP was the recommended treatment for children and adults, in practise, amodiaquine and quinine were sold as often as SP. This may to some extent reflect that amodiaquine was slightly more readily available in drug stores and, according to anecdotal evidence, quinine was popular as it was often regarded a strong and powerful medicine [24]. Overall, it was more likely that a mystery shopper received an antimalarial or even SP in a drug store. However, drug stores did not adhere better to the guidelines than general shops. In part, this may be attributed to the larger choice of products in drug stores. Mere non-availability may also be a reason why no other antimalarials than SP, amodiaquine and quinine were sold, along with the fact that with the cash provided by the researchers, the mystery shoppers would not have been able to purchase expensive drugs such as artemisinin mono therapies or ACT [32].

Altogether, adults would more readily be dispensed an antimalarial than children. This is interesting in the light of findings from a cross-sectional community-survey in which adults would be treated more frequently with shop bought drugs while children were more often brought to a health facility [14]. This may give some indications of provider-side influences on treatment-seeking behaviour.

Treatments for adults were 25% cheaper than treatments obtained for very young children and drug stores were more expensive than general shops. The latter was also found in another study in the same area, where more expensive treatments were obtained from non-governmental organisation (NGO) facilities and drug stores, usually by people from the better-off socio-economic stratum [33].

Private retailers may commonly be perceived as being mainly business-driven in their behaviour. In this study we found that in theory, more than half of all shopkeepers said they would refer severely ill patients and general shopkeepers commonly regarded referral as best option for young children. In practice, 15% (3/20) of drug stores and 31% (25/82) of general shops did not sell any medicines but referred the simulated patients to a higher level of care – although they would have had drugs in their shops. The awareness of shopkeepers that certain cases need to be dealt with at a higher level may be a good entry point for interventions targeted at the retailer level. Several projects targeting private drug retailers, have already counted on the ability and willingness of shopkeepers to refer severe or complicated cases to an appropriate facility [13,34].

### Implications for policy and interventions

The importance of the retail sector as a source of malaria treatment and care complementary to health facility has been recognised internationally [35] and within Tanzania [26]. However, the major concern regarding the private sector has been inadequacy of the treatments offered by often untrained (or not sufficiently trained) shopkeepers [3,34,36]. This issue has re-emerged in the discussions about appropriate delivery channels for ACTs. Defining the role of each type of retailer present in a health system within the frame of their capabilities and the given legal context is an important first step in improving quality and access.

Fully-fledged pharmacies only reach 17% of the Tanzanian population and are hence not sufficient to meet the demand for essential drugs [37]. Part II drug stores which are the largest network of licensed drug-retailers in Tanzania [18] are licensed to sell only OTC drugs, to which none of the recommended antimalarials belongs. Kachur *et al*. showed that patients at drug stores are as likely to be infected with malaria as patients seeking care at health facilities [9]. Considering this demand for antimalarial treatments, there is a need to make efficacious antimalarial drugs available in drug stores. In reality this is usually tolerated by the authorities who recognise the lack of alternatives. In order to improve the quality of services in drug stores, specialised training for drug vendors may be a valid option for improving management of malaria-cases, as has been shown in other areas [12]. The mere definition of educational prerequisites as currently the case for part II shops may only lure health workers away from health facilities to a more profitable business in the retail sector. Yet, training alone is unlikely to improve performance if not coupled with appropriate means of rewarding the shopkeepers for good practices [36,38]. These approaches are combined in a project that upgrades part II shops and potentially general shops to Accredited Drug Dispensing Outlets (ADDO) and that is currently being implemented in selected districts in Tanzania [13,37].

The role of general shops should not be the dispensing of prescription medicines. Yet, due to their importance as easily accessible first contact point for malaria patients, they should not be completely left aside when targeting the private sector. There are several options to strengthen their role in the health sector. Firstly, they could be upgraded to drug retailers (e.g. ADDOs) if appropriately trained, thereby increasing the population coverage with antimalarial providers. Secondly, general shopkeepers could be trained on the appropriate first aid for malaria cases with OTC medicines and subsequent referral to a higher level. Considering that general shops may manage malaria cases only with antipyretics, particularly in places where they are the nearest provider, targeted information or training may decrease the number of inappropriately managed cases at the lowest level. The social pressure exerted on shopkeepers by communities' expectations on their performance should not be under-estimated. In our study, a considerable number of shopkeepers did without business in favour of referring the patient to a drug store or a health facility.

Including all levels of formal and informal health care providers is feasible within the existing legal framework and guided by the national malaria control policy. Alternative approaches including lowest level shops may be a step forward in improving access for people living in remote areas or deprived villages which so far lack any provider of antimalarial medicines [24].

## Conclusion

Private retailers play an important role in the provision of prompt and effective malaria treatment, complementing the services of formal health facilities. Yet, the quality of case-management in the retail sector leaves much room for improvement. Drug stores should be empowered and encouraged to provide correct malaria-treatment with drugs they are legally allowed to dispense. At the same time, the role of general shops as important first contact points for malaria patients needs to be re-considered within the given legal framework.

Interventions on shop-level should consider all types of private retailers. While antimalarial medicines, such as ACTs ought to be dispensed only by qualified personnel, general shopkeepers may acquire sufficient knowledge to properly recognise malaria cases and refer them to a trained provider.

## Competing interests

The authors declare that they have no competing interests.

## Authors' contributions

MWH was responsible for all aspects of the shop census, contributed to the development of the mystery shoppers study, selected the sample, analysed the data together with AD and wrote the manuscript in collaboration with the other authors. JJM prepared the mystery shoppers research plan and data collection activities, and supervised the field-work. CL and BO conceived the research questions and contributed to the design of both studies and the discussion of the manuscript. AM and CM provided support during field-work and contributed to the discussion of the findings. AS and HM contributed to the research questions and the study design. All authors read and approved the final manuscript.

## Pre-publication history

The pre-publication history for this paper can be accessed here:


